# Remarks on Mr. Bellingham's Case of Hydrophobia, with Arguments in Favour of Bleeding in That Disease

**Published:** 1815-03

**Authors:** Thomas Key

**Affiliations:** Fenchurch-street


*For the Medical and Physical Journal.*

### *Remarks on Mr. Bellingham's Case of Hydrophobia, with Arguments in favour of Bleeding in that Disease;* by Mr. T. Key.

FEW diseases have resisted the various endeavours for their cure more than Hydrophobia, and we must, perhaps, wait the event of future research, by dissection, before confidence can be acquired in the remedies to be employed in that formidable complaint. We should, then, with our present limited knowlege, not hastily reject a mode of treatment which, hitherto, appears to have been the most successful. Mr. Bellingham, in the last Number of the Medical and Physical Journal, has related a case which he considers as an individual instance militating against the practice recommended by Dr. Schoolbred. The fatal termination may seem to favour the opinion. The most questionable part of the treatment of that case, is the omission of another copious bleeding at Mr. Bellingham's second visit, an evacuation then as imperiously required as the one he so judiciously procured when he first saw the child. Accelerated arterial action was, by his own statement, proceeding with increased power. The omission of the injections appears to have been the neglect of those who were directed to administer them; it is, however, to be regretted that they were so unfortunately protracted, as, when procured, even in an advanced stage of the disease, each produced its respective relief. I cannot, therefore, admit "that depletion had the fullest and the fairest trial."

Without presuming to discuss the intricate question as to the modus operandi of the hydrophobic poison, from the period of its insertion to that of its visible action on the system, it must be admitted that hydrophobia is a disease of more than ordinary excitement; nor, without assuming the existence of some morbid affection of the brain as the primary cause, can any reasoning illustrate the source of the symptoms, symptoms, or the rapidity with which it proceeds to its termination; and, from the manner in which the functions of that organ appear to be deranged, it is scarcely to be disputed that the morbid affection is inflammation; if so, success in the treatment can only be presumed by depletion, actively and judiciously induced, and permanently secured. The most preferable mode of effecting it must be decided by the practitioner; he should, however, be aware, that, as the period allotted for his exertions is so limited, no portion of it should be misemployed. As to blood-letting, if any general rule will apply, it must be that of pursuing it *ad deliquium;* no given quantity of blood to be taken away can be specified, for, as may be seen by a reference to Mr. Bellingham's case, when, though he took away eleven ounces of blood at the first bleeding, a copious quantity for a child of three years and a half old to lose at one operation, yet, in a very few hours, it was again required. Next to bleeding is the evacuation by the bowels, and should be as rigidly enforced. The remedies usually understood as antispasmodics, and too often employed when convulsions prevail, are in hydrophobia injurious; most of them tend to increase the vascular action of the brain, and all interfere with the alvine evacuations.

Having assumed the opinion that the primary affection in hydrophobia is inflammation of the brain, I deem it irrelevant to go into the inquiry as to its identity with gastritis, or any similar affection; they are secondary, and only mark the progress of the disease. The remarks I have made on Mr. Bellingham's case were unavoidable: at variance with his inference, I may be excused for having availed myself of what was necessary to defend my own; nor are they made with any other view than to support a practice which I do not conceive the unfortunate termination of his case to militate against.

*Fenchurch-street;* THOMAS KEY.

*Jan.* 18, 181[illegible].

---

*For the Medical and Physical Journal.*

